# Positional symmetry of porion and external auditory meatus in facial asymmetry

**DOI:** 10.1186/s40902-015-0033-1

**Published:** 2015-10-01

**Authors:** Ji Wook Choi, Seo Yeon Jung, Hak-Jin Kim, Sang-Hwy Lee

**Affiliations:** Department of Oral and Maxillofacial Surgery, College of Dentistry, Yonsei University, 50 Yonsei-ro, Seodaemun-gu 120-752 Seoul, Korea

**Keywords:** Porion, External auditory meatus, Maxilla, Canting, Facial asymmetry

## Abstract

**Background:**

The porion (Po) is used to construct the Frankfort horizontal (FH) plane for cephalometrics, and the external auditory meatus (EAM) is to transfer and mount the dental model with facebow. The classical assumption is that EAM represents Po by the parallel positioning. However, we are sometimes questioning about the possible positional disparity between Po and EAM, when the occlusal cant or facial midline is different from our clinical understandings. The purpose of this study was to evaluate the positional parallelism of Po and EAM in facial asymmetries, and also to investigate their relationship with the maxillary occlusal cant.

**Methods:**

The 67 subjects were classified into three groups. Group I had normal subjects with facial symmetry (1.05 ± 0.52 mm of average chin deviation) with minimal occlusal cant (<1.5 mm). Asymmetry group II-A had no maxillary occlusal cant (average 0.60 ± 0.36), while asymmetry group II-B had occlusal cant (average 3.72 ± 1.47). The distances of bilateral Po, EAM, and mesiobuccal cusp tips of the maxillary first molars (Mx) from the horizontal orbital plane (Orb) and the coronal plane were measured on the three-dimensional computed tomographic images. Their right and left side distance discrepancies were calculated and statistically compared.

**Results:**

EAM was located 10.3 mm below and 2.3 mm anterior to Po in group I.  The vertical distances from Po to EAM of both sides were significantly different in group II-B (*p* = 0.001), while other groups were not. Interside discrepancy of the vertical distances from EAM to Mx in group II-B also showed the significant differences, as compared with those from Po to Mx and from Orb to Mx.

**Conclusions:**

The subjects with facial asymmetry and prominent maxillary occlusal cant tend to have the symmetric position of Po but asymmetric EAM. Some caution or other measures will be helpful for them to be used during the clinical procedures.

## Background

Porion (Po) is an anatomical landmark point for the craniofacial evaluation. It is defined as the most superior and outer bony surface point of the external auditory meatus and can be called as the anatomical Po [1]. It has been employed to construct the Frankfort horizontal (FH) plane, which has been frequently used as a horizontal reference plane for the anthropometric and the cephalometric analysis since its introduction in 1884 [2].

Meanwhile, Po is frequently overlapped with adjacent anatomical structures on two-dimensional (2D) cephalometric radiographs that it is sometimes difficult to identify this anatomical Po due to its lack of visibility [1]. The head positioning of 2D cephalometric radiographs has been implemented using ear rods of cephalostat [3]. The center point of the radiopacity generated by the ear rods of the cephalostat generally represents the machine Po [4]. Some authors declared that this machine Po can replace the anatomical Po, when the anatomical Po is not identifiable. And the ear rods of cephalostats are located at the external auditory meatus (EAM) that machine Po and EAM can be easily accepted to be identical.

The maxillary skeletal and dental position in relation to the cranium is traditionally reproduced by the facebow registration and the mounting onto the articulator. They have been conventionally used for the variety of dental procedures, including the prosthodontics treatment procedures or the pre-operative diagnosis and wafer fabrication for orthognathic surgery. The facebow device is typically used to reproduce the three-dimensional (3D) position of the maxillary and mandibular dental arch in relation to the cranium [5]. This device also employs the ear rods that are inserted into the EAM.

So, we can consider that the ear rods of the cephalostats in cephalometric machine and those of facebow are technically the same in that they rely on the anatomical position of EAM. In addition, they rely on the same classical assumption that EAM can represent Po with the parallel positioning. Thus, EAM can be meaningful only when Po can be reproduced by EAM. However, we sometimes suffered from the doubt of their parallelism when the occlusal cant or facial midline is different from our clinical understandings. This condition is worse when the patients have the facial asymmetries. We would not be confident to the procedures of putting the ear rods to EAM and/or the positional disparity between Po and EAM.

The procedures of precise diagnosis and proper surgical treatment planning are critically important for patients with facial asymmetry. Those procedures should include the dedication to the underlying cause of the facial asymmetry. However, 2D cephalometric analysis with FH plane and/or the dental model mounting with the facebow transfer frequently fails to provide enough information for the accurate diagnosis and treatment planning. Again, it certainly has to do with the following: (1) the poor manipulation of the ear rods for cephalometrics, facebow, and articulator; (2) the designation of the reference points; or (3) the asymmetric locations of Po and/or EAM.

It is our hypothesis that Po on the temporal part of the cranial bone, and/or EAM by the elastic cartilage part of the outer third ear canal may have an asymmetric position in facial asymmetries. This reasoning is based on the simple fact that the facial asymmetry can be accompanied by varying degrees of cranial base asymmetry [6]. Most facial asymmetries other than craniofacial syndromic anomalies present that the cranial base is symmetrical while facial parts are asymmetrical [7]. But there have been also some studies describing the facial asymmetry concurrent with cranial asymmetry. Hayashi reported that the morphology of the cranial base has an effect on the positions of the maxilla and the mandible [8]. Kim et al. also described that the cranial base volume increased on the non-deviated side in patients with facial asymmetry and mandibular prognathism [9].

3D computed tomography (CT) is now a major imaging tool for craniofacial evaluation and treatment planning. The complex craniofacial structures can be radiographically investigated in various aspects and can be measured more precisely using the 3D CT images [10]. Thus, the identification of Po and EAM is much easier and more accurate on 3D CT reconstructed images, and we wanted to examine our hypothesis with this imaging modality.

Considering all these facts, the right and left symmetrical position of Po and EAM and their parallelism are the basic but essential premise for anthropologic or cephalometric analysis as well as the dental procedures with the facebow-transferred dental model mounting [1, 11]. To the best of our knowledge, there are a few studies about the 3D positional symmetry and parallelism of Po and EAM [12]. Thus, authors wanted to know whether Po and EAM in facial asymmetries have 3D positional symmetries. It will be especially useful for the diagnosis and treatment planning of facial asymmetries with maxillary occlusal cant. This study focused on the evaluation of the positional similarity of Po and EAM in facial asymmetries, and also the investigation of their relationship to the maxillary occlusal cant.

## Methods

The 3D CT reconstruction images from 67 subjects (35 males and 32 females) were included in the present study (Table 1). The 22 subjects (14 males and 8 females) for control group (group I) had normal occlusion and maxillary-mandibular skeletal relationship, with the chin top deviations of less than 3 mm and minimal maxillary occlusal cant (less than 1.5 mm). The 45 subjects (21 males and 24 females) were diagnosed with facial asymmetry with the chin top deviations of more than 5 mm on CT at the department of oral and maxillofacial surgery by one surgeon and were included as facial asymmetry group (group II). Their CT images were same as the subjects of our previous studies with the detailed selection criteria (IRB 2-2011-0016) [13,14]. Written informed consent was obtained from the subjects for the publication of this report and any accompanying images. Subjects with systemic disease, craniofacial anomalies, cleft lip and/or palate, trauma, and previous history of surgery were excluded from the present study.

**Table 1:** Groups and their clinical features for this study

Group	Number	Age	Maxillary dental canting	Chin deviation
Group I (control)	22	23.7 ± 3.4	0.70 ± 0.45	1.05 ± 0.52
Group II (asymmetry)	45	21.1 ± 3.7	2.89 ± 1.88	9.27 ± 3.66
II-A (no canting)	12	21.3 ± 3.1	0.60 ± 0.36	7.83 ± 3.05
II-B (canting)	33	21.0 ± 3.9	3.72 ± 1.47	9.79 ± 3.77
Total	67	21.9 ± 3.8	2.17 ± 1.87	6.57 ± 4.92

Mean ± standard deviation, mm

Their CT scans were acquired with the High-speed Advantage CT scanner (GE Medical System Milwaukee, USA) used with high resolution bone algorithm (200 mA, 120 kV) at 1 s, 1 mm slice thickness, and 512 × 512 pixel reconstruction matrix. Reformatted 3D images were created from the CT scan data and were analyzed using SimPlant software (version 14.0, Materialise NV, Leuven, Belgium).

For the 3D reference planes, anatomical landmark points of cranium, which were already used previously [15, 16], were defined as follows:

1. Foramen cecum (FC): the most anterior and superior point of a pit on the cribriform plate between the crista galli and endocranial wall of frontal bone
2. Center of foramen magnum (CFM): the midpoint of foramen magnum at the level of basion
3. Falx cerebri (FxCe): the point of falx cerebri near bregma on the coronal section
4. Optic canal (Oc): the most superior point of optic canal, both sides
5. Eye ball center (EC): the center point of eye ball in sagittal, axial, and coronal plane, both sides
6. Opisthion (Op): the midpoint on the posterior margin of the foramen magnum

Three craniofacial reference planes were constructed on the 3D skull images for measurements. The midsagittal plane (MSP) was first determined as a plane passing through FxCe, FC, and CFM, which were selected as stable and reliable points in our previous reports [15]. The horizontal orbital plane (Orb) was designed as a horizontal plane above FH plane, being perpendicular to the MSP, and passing through the orbital axis plane with the midpoint of Oc and the midpoint of EC [14, 16]. The frontal plane (Fro) was defined as a coronal reference plane which was perpendicular to MSP and Orb and passing the Op.

And the reference point for Po, EAM, and maxillary first molars (Mx) point was defined and marked on 3D reconstruction image as follows:

1. Po: the most superior and outer point on the ovoid bony contour of the external auditory meatus, both sides (Fig. 1)

**Fig. 1:**
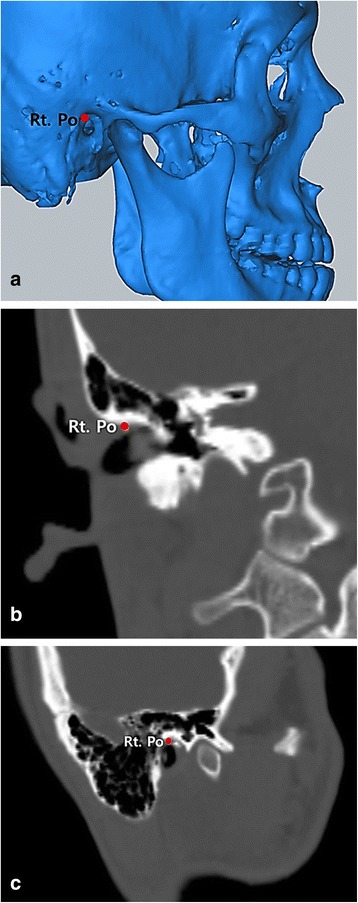
Determination of Po point on 3D CT image. Po was defined as the most superior and outer point on the ovoid bony shadow of the external auditory meatus. **a** Lateral view of 3D reconstruction image. **b** Frontal view of right Po. **c** Lateral view of right Po. *Po* porion
2. EAM: the center point of the most outer round or ovoid shadow of the external auditory meatus, both sides (Fig. 2)

**Fig. 2:**
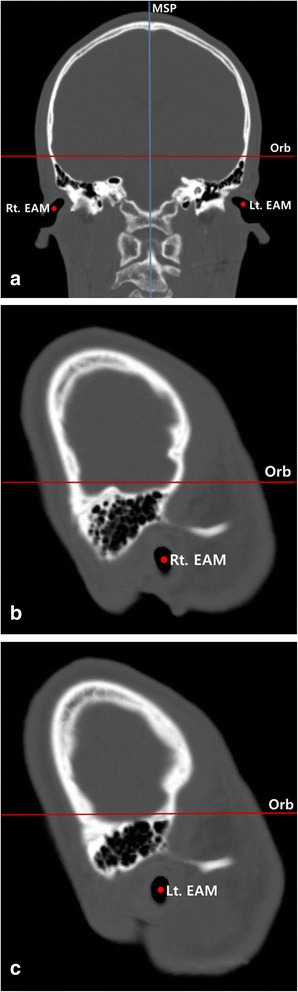
Determination of EAM point on 3D CT image. EAM was defined as a center point of the most outer ovoid soft tissue shadow of the external auditory meatus. **a** Frontal view. **b** Lateral of right EAM. **c** Lateral view of left EAM. *MSP* midsagittal plane, *Orb* orbital plane, *EAM* external auditory meatus
3. Mx: the mesiobuccal cusp tip of maxillary first molar, both sides

**Fig. 3:**
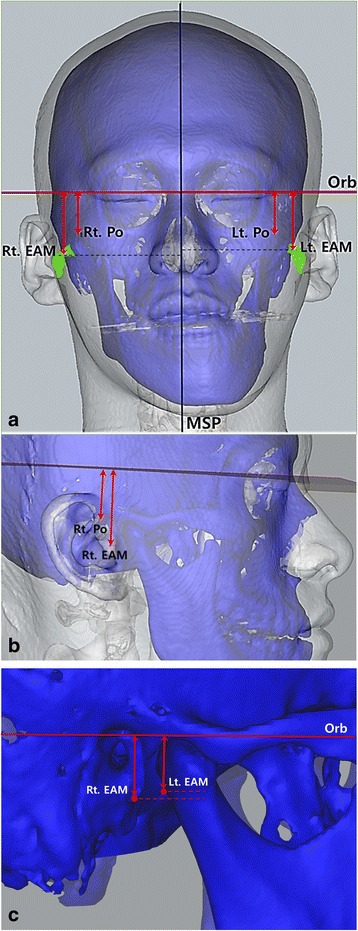
Measurements of vertical distances from Po to Orb/EAM to Orb to indicate the asymmetrical locations of bilateral EAM in facial asymmetry subjects with remarkable maxillary occlusal cant. **a** Frontal view. **b** Lateral view. **c** Lateral oblique view of asymmetric locations of bilateral EAM. *MSP* midsagittal plane, *Orb* orbital plane, *Po* porion, *EAM* external auditory meatus)

The vertical distances from Po to Orb (Po-Orb) and from EAM to Orb (EAM-Orb) were measured on both sides to obtain the vertical distances of Po and EAM (Fig. 3). The horizontal distances from Po and EAM to Fro (Fro-Po and Fro-EAM) on both sides were also measured for horizontal (anteroposterior) locations of Po and EAM. The vertical and horizontal distances between Po and EAM were measured on the right and left side.

The chin top deviation was evaluated by the location of menton from MSP. The maxillary occlusal cant was evaluated by measuring the vertical or horizontal distances from Orb/Fro, Po, and EAM to Mx, and the discrepancies between right and left side were calculated as follows:

1. Vertical discrepancy of Orb-Po or horizontal discrepancy of Fro-Po: the interside discrepancy of the vertical/horizontal distances from Orb/Fro to bilateral Po (right–left)
2. Vertical discrepancy of Orb-EAM or horizontal discrepancy of Fro-EAM: the interside discrepancy of the vertical/horizontal distances from Orb/Fro to bilateral EAM (right–left)
3. Vertical discrepancy of Orb-Mx or horizontal discrepancy of Fro-Mx: the interside discrepancy of the vertical/horizontal distances from Orb/Fro to Mx (right–left)
4. Vertical or horizontal discrepancy of Po-Mx: the interside discrepancy of the vertical/horizontal distances from Po to Mx (right–left)
5. Vertical or horizontal discrepancy of EAM-Mx: the interside discrepancy of the vertical/horizontal distances from EAM to Mx (right–left)

After the 3D image analysis, the subjects were divided into three groups. Group I was defined as the control group to include the normal control subjects without any chin deviation or occlusal cant. Group II (45 subjects with facial asymmetry) were subdivided into two groups, group II-A and group II-B, on the basis of the vertical maxillary occlusal cant (measured as the vertical interside discrepancy of Orb-Mx) (Table 1). Group II-A included 12 subjects (6 males and 6 females) and accounted for 27 % of group II. They had the prominent chin top deviation (more than 5 mm) and minimal maxillary occlusal cant of less than 1.5 mm. Group II-B included 33 subjects (15 males and 18 females) and occupied 73 % of group II. They had the same severe chin top deviation (more than 5 mm) and remarkable maxillary occlusal canting being more than 1.5 mm.

To eliminate statistical confounder by the negative values related to the vertical maxillary cant direction, the 3D images and measurement data were subject to be adjusted to let all subjects have the right downward maxillary cant. The skull images with the left downward maxillary vertical cant were flipped by MSP, to have the changed right and left side. Statistical comparisons of variance and mean values for all three groups were made using one-way ANOVA and post hoc Tukey test by Statistical Package for the Social Sciences (SPSS, Version 21, IBM co.). Paired *t* tests were also used to compare the distances between right and left side, and the maxillary occlusal cants to other measurements.

## Results

We marked and measured the distances for Po and EAM to understand the symmetry of them and their relationship with the maxillary occlusal cant on 3D CT images. Table 1 shows the clinical features of each group. Group I had 0.70 ± 0.45 mm of maxillary occlusal cant and 1.05 ± 0.52 mm of chin top deviation. Group II-A had 0.60 ± 0.36 mm of occlusal cant and 7.83 ± 3.05 mm of chin deviation, while group II-B had 3.72 ± 1.47 mm of maxillary occlusal cant and 9.79 ± 3.77 mm of chin top deviation. The maxillary occlusal cants of group II-A and II-B were statistically significantly different by Student’s *t* test (*p* < 0.001). Meanwhile, the chin top deviation was not significantly different between that of group II-A and II-B (*p* = 0.056).

Table 2 shows that the interside differences of the vertical and horizontal and distances from the reference plane (Orb/Fro) to Po were within the averages of −0.59~0.62 mm, and they were all not significantly different for all three groups (*p* = 0.554 for vertical distances and 0.611 for horizontal distances). Though the detailed data were not presented here, 48 % subjects had vertical Po discrepancies of less than 1.0 mm (59 % in group I, 25 % in group II-A, and 46 % in group II-B). In addition, only 12 % subjects showed more than 3.0 mm of the vertical distance discrepancies for Po (9 % in group I, 17 % in group II-A, and 12 % in group II-B). And 36 % subjects had horizontal discrepancies of Po being less than 1.0 mm, while 21 % subject showed 3 mm or more horizontal discrepancies of Po.

**Table 2:** The interside discrepancies of distances from porion (Po) to orbital plane or frontal plane (Orb or Fro)/external auditory meatus (EAM) to Orb or Fro at the downward-canted (right) and upward-canted (left) side

Discrepancies		Group I	Group II-A	Group II-B	*p* value
Vertical	Orb-Po	0.24 ± 1.78	−0.59 ± 2.73	−0.13 ± 2.13	0.554
	Orb-EAM	0.25 ± 1.88	−0.60 ± 1.84	0.79 ± 1.94	0.099
	*p* value	0.985	0.981	0.001*	
Horizontal	Fro-Po	−0.04 ± 2.18	−0.11 ± 2.30	0.62 ± 3.34	0.611
	Fro-EAM	−0.13 ± 2.87	0.17 ± 2.10	−0.05 ± 2.45	0.755
	*p* value	0.876	0.633	0.110	

Mean ± standard deviation, mm, One way ANOVA and paired *t* test

*Po* porion, *EAM* external auditory meatus, *Orb* orbital plane, *Fro* frontal plane, * denotes statistical difference (*p* < 0.05)

Again, Table 2 shows that the interside differences of the vertical and horizontal distances from the reference plane (Orb/Fro) to EAM were within the averages of −0.60~0.79 mm, and they were all not significantly different for all three groups (*p* = 0.099 for vertical distances and 0.755 for horizontal distances). Though the detailed data were not shown here, 46 % subjects had vertical difference of EAM within the range of −1.0 to 1.0 mm (55 % in group I, 33 % in group II-A, and 46 % in group II-B). And the 12 % subjects showed remarkable vertical EAM discrepancies greater than 3 mm (18 % in group I, 8 % in group II-A, and 12 % in group II-B). And 33 % subjects had horizontal discrepancies of EAM in range of −1.0~1.0 mm, while 19 % subject showed 3 mm or more horizontal discrepancies of EAM.

When we compared the vertical and horizontal distance discrepancies of the right and left side for Orb-Po and Orb-EAM in Table 2, groups I and II-A did not show any significant differences. However, group II-B showed the statistically significant differences for the vertical distance of interside discrepancy for Orb-Po and Orb-EAM (*p* = 0.001).

The results of Table 3 presented that EAM was located 10.35 ± 2.78 mm below and 2.32 ± 2.27 mm anteriorly from Po on the right and left side of group I. And these measurements were almost same for group II, except for the horizontal distances of group II-A (3.74 mm on the right and 3.46 mm on the left side). But the vertical distances between Po and EAM showed significant interside difference in group II-B (*p* = 0.001), with 10.89 mm on the right side and 9.96 mm on the left side. The vertical distance between Po and EAM of the right side was 0.93 ± 1.45 mm greater than that of the left side in group II-B. And the horizontal distances between Po and EAM did not show any significant interside differences for all groups, though that distance interside difference of group II-B reached −0.67 mm (*p* = 0.110).

**Table 3:** Vertical and horizontal distances between porion (Po) and external auditory meatus (EAM) of the downward-canted (right) and upward-canted (left) side

Po-EAM distances		Group I	Group II-A	Group II-B
Vertical	Right	10.35 ± 3.17	10.98 ± 2.32	10.89 ± 2.53
	Left	10.34 ± 2.41	10.99 ± 2.62	9.96 ± 2.21
	Diff	0.01 ± 2.28	−0.01 ± 1.67	0.93 ± 1.45
	*p* value	0.985	0.981	0.001*
Horizontal	Right	2.28 ± 2.19	3.74 ± 2.64	2.38 ± 2.95
	Left	2.37 ± 2.35	3.46 ± 2.08	3.05 ± 2.07
	Diff	−0.09 ± 2.69	0.28 ± 1.96	−0.67 ± 2.36
	*p* value	0.876	0.633	0.110

Mean ± standard deviation, mm, paired *t* test

*Po* porion, *EAM* external auditory meatus, *Diff* difference between the right and left side distance, * denotes statistical difference (*p* < 0.05)

On Table 4, all interside discrepancies of the vertical and horizontal distances for Orb-Mx, Po-Mx and EAM-Mx in group I and group II-A were not statistically different (*p* = 0.633~0.985). However, group II-B had the significantly different vertical measurements for Orb-Mx vs. EAM-Mx and Po-Mx vs. EAM-Mx (*p* = 0.026 and 0.001 each). In addition, all horizontal discrepancies of Fro-Mx, Po-Mx, and EAM-Mx were not significantly different from each other in all groups, and they did not have significant intergroup differences.

**Table 4:** The downward-canted (right) and upward-canted (left) distance discrepancies of Orb-Mx, Po-Mx, and EAM-Mx

Discrepancies		Group I	Group II-A	Group II-B	*p* value
Vertical	Orb-Mx	0.70 ± 0.45	0.60 ± 0.36	3.72 ± 1.47	<0.001
	Po-Mx	0.46 ± 1.89	1.19 ± 2.56	3.85 ± 2.53	<0.001
	EAM-Mx	0.45 ± 1.90	1.20 ± 1.70	2.93 ± 2.35	<0.001
*p* value	Orb-Mx vs. Po-Mx	0.530	0.472	0.718	
	Orb-Mx vs. EAM-Mx	0.538	0.284	0.026*	
	Po-Mx vs. EAM-Mx	0.985	0.981	0.001*	
Horizontal	Fro-Mx	0.33 ± 1.68	−1.93 ± 4.02	−0.08 ± 3.01	0.086
	Po-Mx	0.37 ± 2.51	−1.82 ± 4.26	−0.71 ± 3.24	0.166
	EAM-Mx	0.46 ± 2.66	−2.09 ± 4.22	−0.03 ± 2.67	0.057
*p* value	Fro-Mx vs. Po-Mx	0.938	0.872	0.290	
	Fro-Mx vs. EAM-Mx	0.837	0.785	0.909	
	Po-Mx vs. EAM-Mx	0.876	0.633	0.110	

Mean ± standard deviation, mm, One way ANOVA and paired *t*-test

*Mx* maxillary 1st molar tip, *Po* porion, *EAM* external auditory meatus, *Orb* orbital plane, *Fro* frontal plane, * denotes statistical difference (*p* < 0.05)

## Discussion

Po is a simple anatomical landmark that can be used in anthropometric and cephalometric analysis, and is frequently represented by EAM. If the position of EAM is not parallel with Po, it can make the distorted or inaccurate baseline to construct the reference plane or model mounting. And it can lead to the deep errors for the analysis of facial asymmetry. So, we want to evaluate the positional parallelism or similarity of Po and EAM in facial asymmetries and also to investigate their relationship with the maxillary occlusal cant.

The authors first wanted to construct the reliable horizontal reference planes except FH plane for the verification of FH plane-related reference points in this study. We first established the MSP as a balanced and stable midsagittal plane, based on our previous investigation with 3D CT images of normal subjects and dry skull [15]. The Orb were also designed to construct a horizontal plane, which replicates the visual axis and mimics the natural head position, away from FH plane by previous study [16].

Our first result showed that the vertical discrepancies of Orb-Po and Orb-EAM were significantly different in group II-B (Table 2). But other groups did not show the significant vertical discrepancies for these measurements. Pelo et al. reported a similar result that no subject out of ten subjects had the symmetrical position of bilateral Po and bilateral orbitale [17]. Their discrepancies were significant in severe asymmetry, while the minimal discrepancy was noticed in light asymmetries. So, these results matched exactly with ours to prove that the asymmetry subject may have more chances to have asymmetrical Po.

When we compared the vertical distance difference for three groups for Orb-Mx, Orb-EAM, and Orb-Po, they were close to zero and greater in the order of amount of chin top deviation. It is consistent with the previous researches that the degree of facial asymmetry is increased with greater distance from the cranium [18, 19]. However, the sizes of horizontal discrepancies were not the case. Moreover, the interside discrepancies of the horizontal position for Po and EAM were greater than those of the vertical position, to be judged by the greater standard deviations. This result corresponded well to a report that the horizontal deviations of Po were greater than those of the vertical deviations, regardless of degree of facial asymmetry [12]. But, our result did not show the statistically significant differences for them that we cannot go further that way.

In our investigation, EAM was located about 10.3 mm below Po and approximately 2.3 mm anterior to Po in the normal subjects of group I. Since Po and EAM are located in such a distance, some authors stated that machine Po is unsuitable as a representation of anatomical Po [4, 20]. Our result of this distance between Po and EAM was slightly greater than that from Pancherz et al.’s investigation, which stated that EAM was located more than 9 mm below and 2 mm anterior to Po in 2D lateral cephalographs [4]. This difference of results not only might be brought by the difference between 2D cephalograph and 3D CT, but it also can be related to the age and race of subjects. Their subjects were 11 to 14 years old, but our subjects were adults (21.9 ± 3.8 years old).

Orthognathic patients are likely to have more significant dentofacial morphologic variations than the normal population group had [1]. In our study, group II-B had a statistically significant vertical difference of right and left side Po-EAM (Table 3). This vertical distance between Po and EAM tends to be greater at downward maxillary canted side. The most severe case showed a 5.59-mm difference between right and left distances of Po-EAM.

As Table 4 showed, the interside discrepancies of Orb-Mx, Po-Mx and EAM-Mx were not statistically significantly different from each other in groups I and II-A. It meant that vertical maxillary occlusal cants measured from these reference planes (including orbital horizontal plane, Po, and EAM) are not statistically different from each other in facial symmetries or asymmetries with minimal maxillary cant. But, the group II-B had the significantly different vertical measurements for Orb-Mx vs. EAM-Mx and Po-Mx vs. EAM-Mx (proven by the paired *t* test). The interside discrepancies of Po-Mx and EAM-Mx in group II-B have the average difference of 0.92 mm. The direction of EAM’s cant was same with that of maxillary occlusal cant in group II-B. Though it is not the big value, it can bring about the bad effects for the diagnosis and treatment planning for orthognathic surgery. If it is the case to be measured from EAM, this would decrease the amount of maxillary occlusal cant. Thus, the maxillary occlusal cant may be underestimated during the facebow registration or the model measurement, and in turn, it will cause the insufficient correction of maxillary occlusal cant and the undesirable chin top position during the orthognathic surgery. It also implies that the single measurement of maxillary cant based only on one reference point is likely to be inaccurate especially for the facial asymmetry with the remarkable maxillary cant.

Meanwhile, our investigation showed that the direction of Po’s vertical cant was not significantly related to the direction of maxillary vertical cant for all groups. And the 51.4 % of subjects had the opposite direction of maxillary occlusal and Po’s cant. So, we now believe we can rely on Po with assurance for the horizontal reference point.

Additionally, group II-A had greater horizontal difference of Fro-Mx than group II-B had. We can guess that the maxillary horizontal transposition of group II-A might be greater than group II-B had, and it would also devote to the acceleration of chin deviation. However, our sample size of group II-A was too small to make a statistically significant conclusion. Further studies with more samples will be helpful in the future.

This study has some other limitations. We used a classification on basis of facial asymmetry and vertical maxillary cant, but it did not address the anteroposterior facial deformities such as prognathism or retrognathism and horizontal maxillary occlusal deviation. Further study with more detailed classifications may give rise to more information about the pattern of Po and EAM locations. The evaluation of mediolateral deviations of Po and EAM were also absent in present investigation, that it was insufficient to understand overall 3D asymmetry.

The Po is generally used to locate the FH plane, which is one of the gold standards for the horizontal reference plane for a long time. In spite of this tradition, many researchers have reported that the FH plane displays large individual variation (with the standard deviation ranged 4.02 °–9.1 °) [21]. Currently, a paradigm shift from 2D to 3D imaging requires a new standard for 3D cephalometric analysis [22]. Thus, the evaluation of 3D locations of the traditional reference points including Po and EAM will be a meaningful task.

In summary, Po tends to have symmetrical vertical locations in symmetrical as well as the facial asymmetry subjects. However, EAM can be located significantly asymmetrically in facial asymmetries with prominent maxillary occlusal cant. The possible asymmetry of EAM should be considered for the diagnosis and measurement of maxillary cant for facial asymmetry.

## Conclusions

1. The vertical cants of EAM and Po were significantly different in facial asymmetries with prominent maxillary occlusal cant.
2. The EAM was located 10.3 mm below and 2.3 mm anterior to Po in normal subjects.
3. The vertical distance of Po-EAM was significantly greater at downward-canted side in facial asymmetries with prominent maxillary occlusal cant.
4. For facial asymmetry subjects with remarkable maxillary occlusal cant, the vertical maxillary cant measured from EAM was significantly smaller than those measured from Po or Orb.

These results indicate that the subjects with facial asymmetry and prominent maxillary occlusal cant tend to have the symmetric positions of Po but asymmetric location of EAM.

## References

[1] Zebeib AM, Naini FB (2014). Variability of the inclination of anatomic horizontal reference planes of the craniofacial complex in relation to the true horizontal line in orthognathic patients. Am J Orthod Dentofacial Orthop.

[2] Naini FB (2013). The Frankfort plane and head positioning in facial aesthetic analysis-the perpetuation of a myth. JAMA Facial Plast Surg.

[3] Katsumata A, Fujishita M, Maeda M, Ariji Y, Ariji E, Langlais RP (2005). 3D-CT evaluation of facial asymmetry. Oral Surg Oral Med Oral Pathol Oral Radiol Endod.

[4] Pancherz H, Gokbuget K (1996). The reliability of the Frankfort horizontal in roentgenographic cephalometry. Eur J Orthod.

[5] Wolford LM, Galiano A (2007). Simple and accurate method for mounting models in orthognathic surgery. J Oral Maxillofac Surg.

[6] Forsberg CT, Burstone CJ, Hanley KJ (1984). Diagnosis and treatment planning of skeletal asymmetry with the submental-vertical radiograph. Am J Orthod.

[7] Baek SH, Cho IS, Chang YI, Kim MJ (2007). Skeletodental factors affecting chin point deviation in female patients with class III malocclusion and facial asymmetry: a three-dimensional analysis using computed tomography. Oral Surg Oral Med Oral Pathol Oral Radiol Endod.

[8] Hayashi I (2003). Morphological relationship between the cranial base and dentofacial complex obtained by reconstructive computer tomographic images. Eur J Orthod.

[9] Kim SJ, Lee KJ, Lee SH, Baik HS (2013). Morphologic relationship between the cranial base and the mandible in patients with facial asymmetry and mandibular prognathism. Am J Orthod Dentofacial Orthop.

[10] Okumura H, Chen LH, Tsutsumi S, Oka M (1999). Three-dimensional virtual imaging of facial skeleton and dental morphologic condition for treatment planning in orthognathic surgery. Am J Orthod Dentofacial Orthop.

[11] Ellis E, Tharanon W, Gambrell K (1992). Accuracy of face-bow transfer: effect on surgical prediction and postsurgical result. J Oral Maxillofac Surg.

[12] Kim MG, Lee JW, Cha KS, Chung DH, Lee SM (2014). Three-dimensional symmetry and parallelism of the skeletal and soft-tissue poria in patients with facial asymmetry. Korean J Orthod.

[13] Lee SH, Kil TJ, Park KR, Kim BC, Kim JG, Piao Z, Corre P (2014). Three-dimensional architectural and structural analysis—a transition in concept and design from Delaire’s cephalometric analysis. Int J Oral Maxillofac Surg.

[14] Park KR, Park HS, Piao Z, Kim MK, Yu HS, Seo JK (2013). Three-dimensional vector analysis of mandibular structural asymmetry. J Craniomaxillofac Surg.

[15] Kim HJ, Kim BC, Kim JG, Zhengguo P, Kang SH, Lee SH (2014). Construction and validation of the midsagittal reference plane based on the skull base symmetry for three-dimensional cephalometric craniofacial analysis. J Craniofac Surg.

[16] Kang YH, Kim BC, Park KR, Yon JY, Kim HJ, Tak HJ (2012). Visual pathway-related horizontal reference plane for three-dimensional craniofacial analysis. Orthod Craniofac Res.

[17] Pelo S, Deli R, Correra P, Boniello R, Gasparini G, Moro A (2009). Evaluation of 2 different reference planes used for the study of asymmetric facial malformations. J Craniofac Surg.

[18] Padwa BL, Kaiser MO, Kaban LB (1997). Occlusal cant in the frontal plane as a reflection of facial asymmetry. J Oral Maxillofac Surg.

[19] Peck S, Peck L, Kataja M (1991). Skeletal asymmetry in esthetically pleasing faces. Angle Orthod.

[20] Ricketts RM (1981). Perspectives in the clinical application of cephalometrics: the first fifty years. Angle Orthod.

[21] Madsen DP, Sampson WJ, Townsend GC (2008) Craniofacial reference plane variation and natural head position. Eur J Orthod 30(5):532-540 10.1093/ejo/cjn031 18632837

[22] Gateno J, Xia JJ, Teichgraeber JF (2011). New 3-dimensional cephalometric analysis for orthognathic surgery. J Oral Maxillofac Surg.

